# Morphology and Properties of Poly(2,6-dimethyl-1,4-phenylene oxide)/Polyamide 11 Hybrid Nanocomposites: Effect of Silica Surface Modification

**DOI:** 10.3390/ma15103421

**Published:** 2022-05-10

**Authors:** Regina Jeziorska, Agnieszka Szadkowska, Maciej Studzinski

**Affiliations:** Lukasiewicz Network—Industrial Chemistry Institute, Rydygiera 8, 01-793 Warsaw, Poland; agnieszka.szadkowska@ichp.pl (A.S.); maciej.studzinski@ichp.pl (M.S.)

**Keywords:** nanocomposites, silica, morphology, dynamic mechanical analysis, mechanical properties, thermal properties

## Abstract

Poly(2,6-dimethyl-1,4-phenylene oxide)/polyamide 11 (PPO/PA11 80/20) blend filled with neat (SiO_2_) or modified silica having amine functional groups (A-SiO_2_) was melt mixing in a twin-screw extruder. The silica was prepared by the sol–gel process. SEM shows that, with increasing A-SiO_2_ content from 1 to 5 wt.%, the morphology of PPO/PA11blend changed from droplet matrix to co-continuous with phase inversion. The phase inversion was also observed for 5 wt.% of neat silica, but the droplet-matrix structure was retained. The overall rheological and mechanical properties improvement of the A-SiO_2_-filled composites in comparison with the unfilled blend and neat silica counterpart was drastic, especially in terms of viscosity and stiffness. A-SiO_2_ improved PPO and PA11 miscibility and reduced the crystallinity of PA11, without affecting the T_c_, owing to the compatibilization effect. On the other hand, neat silica slightly increased the crystallinity of PA11 and decreased the crystallization temperature of PA11 and the glass transition temperature of PPO as a result of its plasticization.

## 1. Introduction

Over the last few decades, polymer blends have been extensively studied because they offer low-cost new polymers with enhanced properties. The majority of multicomponent polymer systems are two-phase blends that display advantages over single-phase systems [1,2,3,4]. The effect of nanofillers on the morphology and properties of polymer blends has gained great attention since they improve physical properties [5,6,7].

Nanofillers such as organoclays and nanosilica shift the droplet-matrix morphology toward a finer dispersion of the minor phase. The key to explaining this phenomenon seems to be the selective localization of nanoparticles in one of the phases, usually in the matrix or the interphase [8,9]. In some cases, they promote the formation of co-continuous structures and also can control blends morphology [10,11]. Compared with the traditional filler-reinforced systems, the improved properties of polymer nanocomposites are mainly due to the stronger interfacial interaction that occurs between the polymer matrix and nanoparticles [12,13]. 

Poly(2,6-dimethyl-1,4-phenylene oxide) (PPO) is one of the most important engineering polymers with high strength, excellent heat resistance, and high transition glass temperature. However, due to the high processing temperature (>280 °C), PPO is used as a component of polymer blends, especially with polystyrenes and polyamides, giving a wide range of engineering plastics with excellent mechanical, dielectric, and chemical properties. Modified PPO is used as components of electronic devices, thermally sterilized surgical instruments, pumps, meters, vehicle mechanics, and household appliances, especially those working at high temperatures and in contact with hot water [14,15].

Epoxycyclohexyl polyhedral oligomeric silsesquioxane (POSS) was added to PPO/PA6 blends, and the obtained composites with co-continuous morphology showed better mechanical properties than those with droplet-matrix morphology. Moreover, epoxycyclohexyl POSS acts as a chain extender and a crosslinking agent for PA6 [16]. Similar results were obtained for PPO/PA6/organically modified montmorillonite composites [17]. Adding carbon nanotubes with different functional groups to PPO/PA6 60/40 blend increased the tensile modulus and storage modulus of the blend, while its tensile strength slightly decreased. The phase structure of the blend changed from sea-island to co-continuous for hydroxylated and amino-functionalized nanotubes. However, the incorporation of carboxylated nanotubes did not change the blend morphology due to the strong aggregation of the nanofiller [18]. 

Blends of PPO and fully bio-based but non-biodegradable polyamide 11 (PA11) are of interest as high-performance materials with great environmental benefits. PPO offers high dimensional and thermal stability, and PA11 shows high chemical and UV resistance, and low melt viscosity [18,19]. It is well known that PPO is incompatible with polyamides, which results in deterioration in impact and tensile strength [14,16]. This is due to poor interfacial adhesion between the dispersed phase and the continuous matrix, which leads to rapid initiation and crack growth [20,21,22]. 

PA11 is synthesized from natural castor oil. It is characterized by a lower melting temperature and density, compared with other polyamides (PA 6.6 or 10.10) [23]. Recent results show that PA11 forms a good interface with flax fibers [24,25]. Gourier and Bourmaud studied the recycling stability of unidirectional flax-fiber-reinforced PA11 in comparison to PP/PP-g-MA/flax [26]. The incorporation of functionalized halloysite in the PA11/SEBS-g-MA 85/15 blend significantly improved toughness and thermal properties without affecting PA11 crystallization. This was due to the good stress transfer from the matrix to the functionalized halloysite agglomerates surrounded by SEBS-g-MA [27]. Montmorillonite, owing to the reinforcing effect, significantly increased the stiffness of polyamide 11 [28].

In our previous research [29], glycidyl methacrylate grafted ethylene-*n*-octene copolymer (GEOC) was used as an effective compatibilizer for PA11/PPO 80/20 blend, with substantial improvement in impact strength. In addition, amine-functionalized silica (A-SiO_2_) was used to control the morphology of the PA11-rich PA11/PPO blend [11]. Functional groups from A-SiO_2_ and GEOC reacted with the terminal end groups on PA11, generating covalent bonds between them, as confirmed by the gel content tests. Despite the lower content, PPO formed a continuous phase, and PA11- a dispersed. Both compatibilizers changed the blend morphology from droplet matrix to co-continuous. The greatest size reduction in both phases, reflecting the highest impact toughness, was observed for 3 wt.% of A-SiO_2_ content. At 5 wt.% of silica, phase inversion was observed with the reappearance of the droplet structure [11]. As expected, the blends with co-continuous structure showed better mechanical properties than those with droplet-matrix morphology. It seems interesting to study the influence of modified silica on the morphology and properties of the PPO/PA11 blend, in which PA11 is a minor phase. To the best of our knowledge, the PPO-rich PPO/PA11 blend compatibilized with silica having amine-functional groups has not yet been studied.

In this study, the effect of amine-functionalized spherical nanosilica (A-SiO_2_) on the morphology, tensile, flexural, and impact properties, as well as dynamic mechanical properties, melting, and crystallization behaviors of the PPO/PA11 80/20 blend, was investigated in detail. To achieve our aim, PPO/PA11/silica nanocomposites at two different loadings of silica (1 and 5 wt.%) were obtained by melt-compounding using a co-rotating twin-screw extruder. The properties were analyzed using several techniques, i.e., scanning electron microscopy (SEM), dynamic-mechanical thermal analysis (DMTA), and differential scanning calorimetry (DSC). Moreover, the properties of the composite with 5 wt.% of modified silica were compared with those of the one with 5 wt.% unmodified silica.

## 2. Experimental

### 2.1. Materials and Processing

Amorphous poly(2,6-dimethyl-1,4-phenylene oxide) (PPO), Noryl V0150B, with MFR 4 g/10 min (measured at 300 °C and 5 kg), was purchased from Sabic GE Plastics Co. (Pittsfield, MA, USA). Biobased semicrystalline polyamide 11 (PA11), Rilsan, with MFR 22 g/10 min (measured at 235 °C and 10 kg), was supplied by Arkema France. Neat (SiO_2_) and modified silica having 0.35 wt.% of amine functional groups (A-SiO_2_), with an average diameter of 30 nm and specific surface area of 274.4 m^2^/g, were prepared by the reported sol–gel method [30,31,32] and used at concentrations of 1 and 5 wt.%. 

### 2.2. Silica Preparation and Characterization

Neat (SiO_2_) and modified silica having amine functional groups (A-SiO_2_) were synthesized using the procedure published elsewhere [30,31,32]. Briefly, ethyl alcohol, aqueous ammonia, and distilled water were mixed. Then, tetraethoxysilane (TES 28, Wacker Chemie, Munich, Germany) was added and stirred for 2 h. **γ**-Aminopropylotriethoxysilane (Momentive Performance Materials, Waterford, NY, USA) was added to the reaction mixture when the pH was in the range of 7.5−10.8, and stirring was continued for 1 h. The obtained silica sol was dried in an oven at 50−90 °C for 2 h.

**Figure 1:**
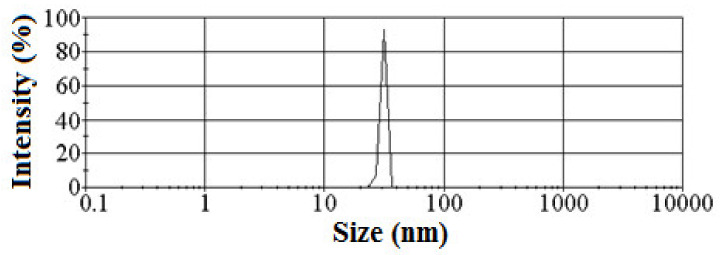
A-SiO_2_ particle size distribution with an average size of 30 nm.

Particle size and particle size distribution of the resulting sol were measured by photon correlation spectroscopy (PCS), using a Malvern apparatus (Zetasizer Nano ZS, Bedford Hills, NY, USA). The monomodal particle size distribution and very low dispersion of particle size were observed for homogeneous sol of amine-functionalized silica (Figure 1). The process allows silica particles to be obtained with almost uniform particle size, relating to the selection of the process parameters.

**Figure 2:**
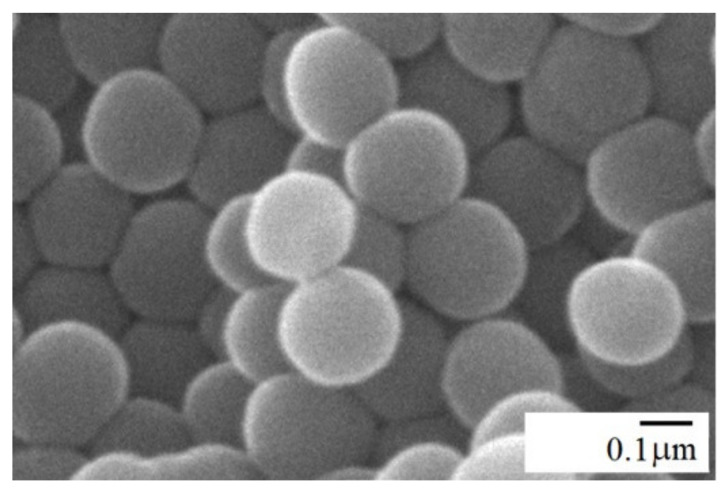
SEM micrograph of A-SiO_2_ particles with an average size of 30 nm.

The specific surface area of silica nanoparticles was measured with the BET-N2 sorption method, using a Gemini 2370 V.302, Norcross, GA, USA, apparatus. The Kjeldahl method based on nitrogen content measurement was used to determine the content of amine groups. The morphology of neat and amine-functionalized silica was studied using a Jeol JSM-6490LV, JEOL, Tokyo, Japan, scanning electron microscope (SEM), operating at an accelerating voltage of 15 kV. Spherical shapes with a uniform size of A-SiO_2_ particles can be observed in the SEM micrograph presented in Figure 2.

### 2.3. Composites Preparation

Prior to mixing, the poly (2,6-dimethyl-1,4-phenyleneoxide) (PPO) and polyamide 11 (PA11) were dried at 85 °C under vacuum for 12 h to remove moisture. All the composites were prepared using a co-rotating 25 mm twin-screw extruder (L/D = 51, KraussMaffei Berstorff, Hanover, Germany) with a rotational speed of 200 rpm according to the procedure published elsewhere [33]. Separate gravimetric feeders were used for PPO, PA11, and silica (SiO_2_ or A-SiO_2_). A very efficient vacuum was applied in the decompression zone. The barrel temperature was set from 215 to 270·°C. After compounding, the material was extruded from the die with two cylindrical nozzles of 4 mm diameter and then rapidly cooled in a water bath and pelletized with an adjustable rotating knife into 4 mm pellets. The composites were injection molded at 270−285 °C using an Arburg 420 M single screw injection machine (Allrounder 1000-250, ARBURG, Loßburg, Germany) to obtain samples for SEM and mechanical tests. The mold temperature was 70 °C.

### 2.4. Methods

The gel content was determined as follows: A 2 g sample was dissolved in 50 mL of chloroform at room temperature. The soluble part was removed by filtration until the deposition of chloroform in the solution was detected by adding excess acetone. Thereafter, the insoluble component was dried and then dissolved in 50 mL of nitric acid at room temperature for 4 h. The soluble portion was removed by filtration until deposition in the nitric acid solution could not be detected by the addition of excess alcohol. The insoluble gel was washed well with alcohol, dried, and weighed. The percentage of insoluble gel was defined as the gel content.

The morphology and distribution of the silica particles in the PPO/PA11 matrix were characterized using a Joel JSM 6100, JEOL, Toyko, Japan, scanning electron microscope (Japan). The samples were etched with chloroform and nitric acid, good solvents for PPO and PA11, respectively, before observation. The etch time was 5 h at room temperature. After etching the samples were cleaned in distilled water and acetone and then dried. The impact fracture surfaces were coated with a thin gold film to avoid charging and to increase image contrast. 

Oscillatory rheological measurements were performed using a rotational rheometer (Rheometrics RDS 2, Rheometric Scientific Inc., Piscataway, NJ, USA) equipped with 25 mm diameter parallel plates at 270 °C and frequency range from 1 to 1000 rad/s. Complex viscosity η* was measured in the frequency sweep experiments. 

Dynamic mechanical analysis (DMTA) was conducted on a Rheometrics RDS 2 dynamic analyzer (Piscataway, NJ, USA), with a specimen dimension of 38 × 10 × 2 mm, prepared by injection molding. The torsion method was used at a frequency of 1 Hz, at a strain level of 0.1% in the temperature range of −150 to 200 °C, and heating rate of 3 °C/min. 

Tensile and flexural properties were studied on an Instron 5500R universal testing machine (Wycombe, UK), according to ISO 527 and ISO 178, respectively. The crosshead speeds for tensile and flexural tests were 5 and 2 mm/min, respectively. The gage length for tensile tests was 50 mm. 

Notched Charpy impact tests (ISO 179) were performed using a Zwick impact tester. All tests were carried out at room temperature. Five measurements were conducted for each data point in all mechanical property tests. 

Thermal properties were evaluated by differential scanning calorimetry (DSC) (Mettler-Toledo, Im Langacher, Switzerland) at a heating rate of 10 °C/min, in a nitrogen atmosphere, with a scan range of temperature from room temperature to 300 °C. Then, the samples were held at 300 °C for 5 min to ensure an identical thermal history and subsequently cooled to room temperature. Finally, samples were heated again to 300 °C. Crystallization temperature (T_c_) was collected from the cooling cycle; meanwhile, melting temperature (T_m_), melting enthalpy (ΔH_m_), and glass transition temperature (T_g_) were carried out from the second heating cycle. Melting enthalpies were calculated considering the filler weight fraction. The degree of crystallinity (X_c_) was calculated from the melting enthalpy results (ΔH_m_) of each sample using Equation (1), where ΔH_m_ and $\Delta H_{m}^{o}$ are the enthalpies of fusion for composites and 100% crystalline PA11 (189 J/g), respectively [34]. W_PA11_ is the weight fraction of PA11 in the samples.

(1) $$X_{c}=\frac{\Delta H_{m}}{w_{\mathrm{PA}11}\Delta H_{m}^{o}}\cdot 100\;\%$$

## 3. Results and Discussion

### 3.1. Graft Copolymer Formation

**Figure 3:**
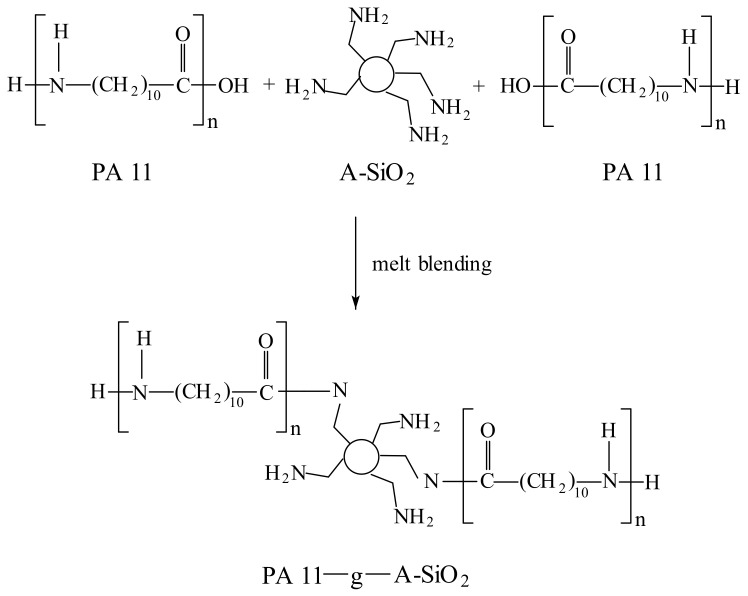
Scheme of the in situ compatibilization.

The amount of in situ formed PA11–*g*–A-SiO_2_ was calculated by the gel content test (Table 1). The samples were successively extracted by chloroform and then by nitric acid. The residue, insoluble in both solvents, was considered a mixture of A-SiO_2_ and A-SiO_2_-grafted PA11. The reactions between amine functional groups of silica and carboxyl groups of PA11 were obvious, as the gel content of the composites increased with increasing A-SiO_2_ content. At A-SiO_2_ content of 5 wt.%, most of the PA11 molecules gelled, which indicates a higher melt viscosity of the PA11 phase and confirms the reaction between A-SiO_2_ and PA11. Figure 3 shows the in situ compatibilization of the PPO/PA11 blend, where part of A-SiO_2_ particles formed graft copolymer with PA11, which can efficiently control the phase morphology of the blend during melt mixing. These results are consistent with our previous study on the PPO/PA11 blend, in which PPO was a minor phase [11]. 

### 3.2. Morphology

**Figure 4:**
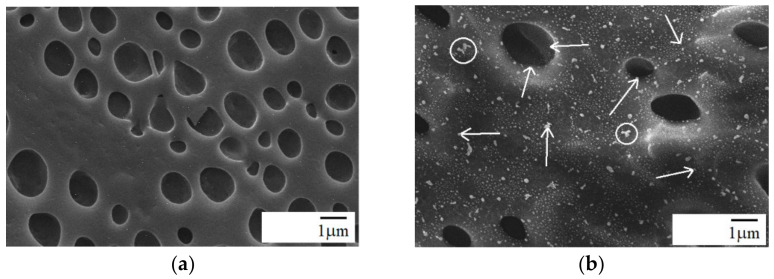
Chloroform etched SEM micrographs: (**a**) PPO/PA11 80/20 blend and (**b**) PPO/PA11/1 wt.% A-SiO_2_ composite; arrows—silica particles, circles—silica agglomerates; all images were reported at scales of 1 µm.

**Figure 5:**
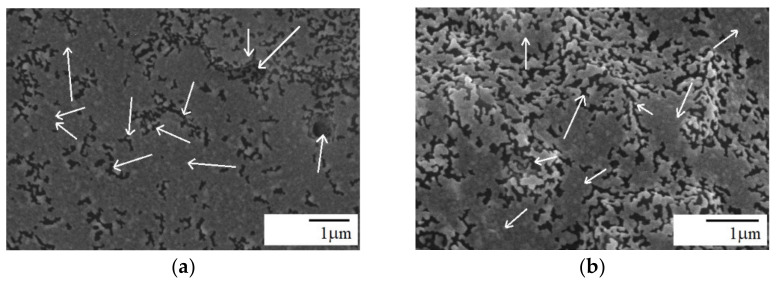
Nitric-acid-etched SEM micrographs of PPO/PA11/A-SiO_2_ 80/20/5 composite: (**a**) low magnification, (**b**) high magnification; arrows—silica particles; all images were reported at scales of 1 µm.

**Figure 6:**
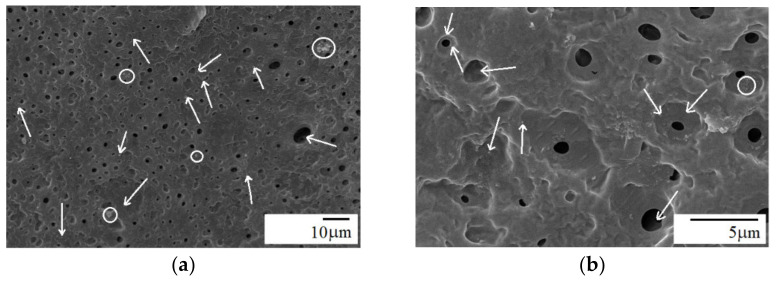
Nitric-acid-etched SEM micrographs of PPO/PA11/SiO_2_ 80/20/5 composite: (**a**) low magnification, (**b**) high magnification; arrows—silica particles, circles—silica agglomerates.

The cross-sectional images of PPO/PA11/silica composites are presented in Figure 4, Figure 5 and Figure 6. Figure 4 shows chloroform etched SEM micrographs of PPO/PA11 80/20 blend and PPO/PA11/A-SiO_2_ 80/20/1 composite, where the black domains indicate the PPO phase etched by chloroform. Indeed, PPO with much higher melt viscosity than PA11 tends to coalesce during melt mixing [22]. It is clear from Figure 4a that the PPO/PA11 80/20 blend showed a typical droplet-matrix morphology, with a dispersed PPO phase and continuous PA11 phase. Uniform dispersion of the holes corresponding to the extracted PPO was observed. However, many of them were larger than 1 µm. It was reported previously that PPO/PA11 20/80 blend also exhibited droplet-matrix structure. However, in this case, PPO formed a continuous phase, and PA11 dispersed [11]. 

**Figure 7:**
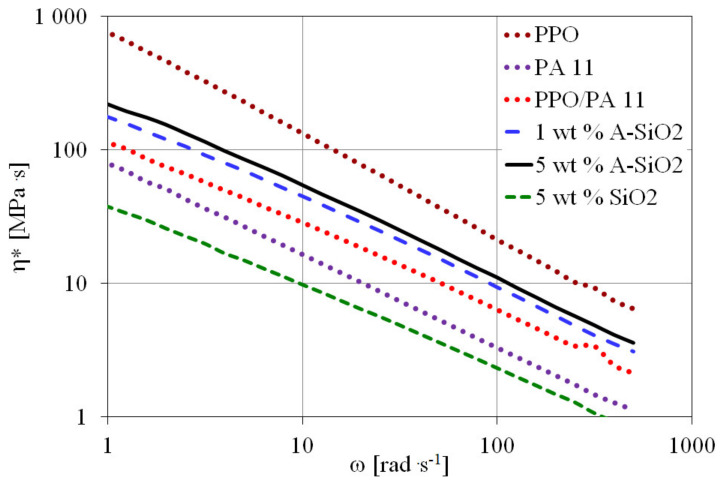
Complex viscosity versus frequency for PPO, PA11, and PPO/PA11/silica composites differing in silica type and loading at 270 °C.

Droplet-matrix structure of the blend appeared relatively unchanged after adding 1 wt.% of A-SiO_2_ (Figure 4b). Homogeneous and uniform dispersion was observed for the silica particles with very few aggregates. The modified silica was mainly located in the PA11 phase and over the interface, due to the reaction of the amine group in the silica with the end groups of PA11 (-COOH). However, very few A-SiO_2_ particles could also be observed in the PPO phase (Figure 4b). The fine dispersion of modified silica particles in PA11 may be the result of a good affinity between A-SiO_2_ and the PA11 matrix. On the other hand, the incorporation of 5 wt.% A-SiO_2_ transformed the droplet morphology into co-continuous with phase inversion, as shown in Figure 5. This is because a portion of loaded A-SiO_2_ was dispersed in the PPO phase since the PA11 continuous phase could not accommodate more A-SiO_2_ particles. This decreased the viscosity mismatch between the two phases, increasing PA11 matrix viscosity; thus, the A-SiO_2_-rich PPO phase was completely elongated, forming an interconnected structure (Figure 5). As a result, the droplet dispersed morphology changed to co-continuous [16]. The results are consistent with the literature data for nanotube-filled PS/PA6 and PPS/PA66 blends [35,36], as well as for PPO/PA6 blend with organically modified clay [17]. Increasing A-SiO_2_ content from 1 to 5 wt.% further reduced the mobility of the interface and increased the viscosity of the PA11 phase (Figure 7). The uniform dispersion with only a few aggregates of silica particles could be seen in the composite containing 5 wt.% A-SiO_2_ (Figure 5b), suggesting strong links. This phenomenon is most probably attributed to the extremely high surface activity of the silica, and the particles consequently have a tendency to aggregate tightly, creating micron-sized silica clusters, especially at higher concentrations [31,37]. 

It is clear from Figure 6 that the addition of 5 wt.% neat silica caused a drastic change in morphology with phase inversion, whereas PPO formed the continuous phase, whereas PA11 and silica formed the dispersed phase. Conversely, neat silica was mainly located in the PPO phase and over the interface. This decreased the viscosity mismatch between the two phases, decreasing PPO viscosity; thus, the SiO_2_-rich PPO phase was completely elongated, forming a continuous phase (Figure 6). As a result, phase inversion was observed [16]. However, many agglomerates of SiO_2_ particles could be observed in the PPO continuous phase. The observed morphology is consistent with the rheological behavior of the composite (Figure 7). A similar trend in morphology was observed for PA11-rich PPO/PA11 blend containing 5 wt.% A-SiO_2_ [11]. 

#### Dynamic Viscosity

The morphology of an incompatible polymer blend is closely related to its rheological behavior. The complex viscosity (η*) versus frequency for PPO/PA11/silica composites and their blend components (PPO and PA11) at 270 °C are shown in Figure 1. All molten polymers showed non-Newtonian flow behavior. The values of the complex viscosity of the PPO/PA11/A-SiO_2_ composites were significantly higher than those of the PPO/PA11 blend. The viscosity increased with increasing A-SiO_2_ content. The reason is that A-SiO_2_ acts as a chain extender or a crosslinking agent for PA11, restricting the movement of PA11 chains and leading to higher viscosity [11]. A similar increase in viscosity was also observed for PPO/PA6/POSS composites [16] and functionalized zirconium-phosphate-filled PA46/PPO blend [7]. The SEM observation showed that A-SiO_2_ was located preferentially in PA11. However, at higher content, A-SiO_2_ was distributed in both phases, as well as along the interface. Therefore, A-SiO_2_ mainly changed the rheological behavior of PA11 but could also affect that of PPO in the composites, especially at higher A-SiO_2_ content. The morphological change in PPO/PA11/A-SiO_2_ composites can be mainly attributed to the effect of A-SiO_2_ on the melting behavior of PA11. 

Figure 7 shows a very unusual phenomenon in which the viscosity of PPO/PA11/SiO_2_ was much lower than that of the PPO/PA11 and even PA11. The lowest viscosity observed is probably due to the plasticization of PPO by neat silica. A similar effect was also observed for HDPE/fumed silica composites [38]. Moreover, the phase inversion can also play an important role, because when neat silica is used, PA11 forms a dispersed and not a continuous phase, as in the case of using modified silica. The viscosities of PPO/PA11/silica composites and their blend components at 100 rad s^−1^ and 270 °C are shown in Table 2.

### 3.3. Dynamic Mechanical Analysis

**Figure 8:**
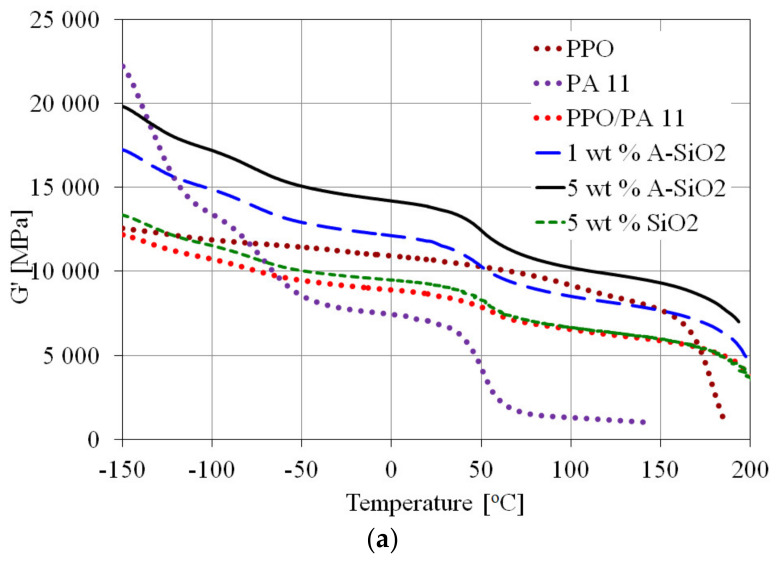

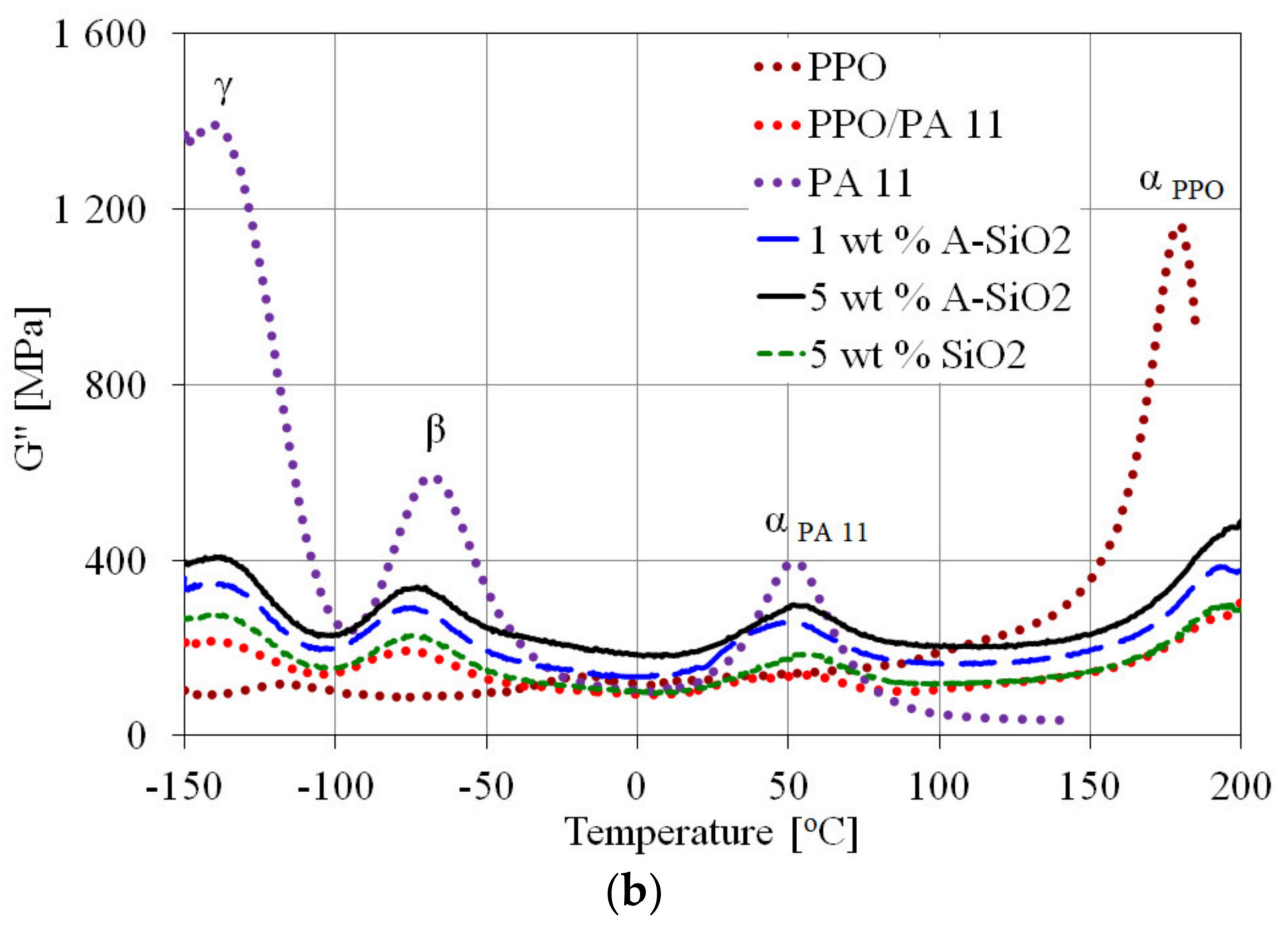
Storage moduli G′ (**a**) and loss moduli G′ (**b**) of PPO, PA11, and PPO/PA11/silica composites as functions of temperature.

The storage moduli (*G*′) of PPO, PA11, and PPO/PA11/silica composites are presented in Figure 8a as a function of temperature. The typical behavior of the storage modulus was observed with three confined regions—namely, the glassy region, the glass transition region, and the rubbery region—corresponding to the relaxation of PA11 and PPO chain segments, respectively. The storage modulus of PPO dramatically decreased when the temperature was higher than 175 °C. On the other hand, three rapid reductions of storage modulus corresponding to the chain segments relaxation were observed in PA11. PPO/PA11 showed a lower storage modulus than PPO. However, the blend exhibited higher storage modulus in the glassy and glass transition regions, and lower in the rubbery region, compared with neat PA11 (Figure 8a). As expected, the storage modulus significantly increased with increasing A-SiO_2_ content, which is caused by the restriction of PA11 and PPO chain segments motions. The enormous improvement in stiffness confirms the strong interactions between the amine group in A-SiO_2_ and the carboxyl group in PA11. Similar results were reported for functionalized carbon nanotubes filled with PPO/PA6 blends [18]. On the other hand, adding 5 wt.% of SiO_2_ resulted in a much lower storage modulus than that of the composites with A-SiO_2_. Moreover, the storage modulus of the PPO/PA11/SiO_2_ composite was only slightly higher in the rubbery and glass transition regions, compared with the PPO/PA11 blend. This can be explained by both the effects of partial plasticization of PPO on neat silica and the good miscibility between SiO_2_ and PPO. The results correspond to the rheological behavior (Figure 7) of the composites and structural change observed by SEM (Figure 6). 

Figure 8b shows the loss moduli (G′′) as a function of temperature for PPO, PA11, and PPO/PA11/silica composites. The α relaxation peak, commonly referred to as the glass transition temperature (T_g_) [11,29,39], was observed at 52 °C for PA11 and 178 °C for PPO. The T_g_ of the blend was 5 °C lower and 13 °C higher than that of PA11 and PPO, respectively. The β relaxation was observed as a weak maximum in loss modulus for PPO, while PA11 and PPO/PA11 showed a clear single β relaxation peak. The β relaxation is caused by movements of amide polar groups of polyamide in the interfacial region and is attributed to glass transition [40]. The T_β_ of PPO/PA11 was 8 °C and 59 °C lower than that of PA11 and PPO, respectively. The γ relaxation is associated with a single relaxation process, predominantly of amorphous origin. The T_γ_ of the blend was observed at −118 °C for PPO and −140 °C for PA11 (Figure 8b, Table 3), with a corresponding decrease in storage modulus (Figure 8a). The PPO/PA11 showed Tγ to be 3 °C higher and 18 °C lower than that of PA11 and PPO, respectively. 

The glass transition temperatures of PA11 and PPO phases increased with increasing A-SiO_2_ content. Indeed, the addition of 5 wt.% of A-SiO_2_ resulted in 9 °C higher T_α PA11_ and 6 °C higher T_α PPO_, compared with PPO/PA11. This reflects the restriction of the polymer chains motion induced by silica and indicates effective interfacial interaction between amine-functionalized silica and PA11, which is consistent with the reported literature [40,41,42]. As expected, the neat-silica-filled PPO/PA11 blend showed also a higher glass transition temperature. However, T_α PPO_ was slightly lower, whereas T_α PA11_ was slightly higher than those of the modified silica-filled PPO/PA11 blend. 

Moreover, the intensity of α relaxation peaks observed in G′′ curves increased with A-SiO_2_ loading, which indicates improved stiffness. However, the intensity of peaks of composite with neat silica was extremely lower than that with the same loading of A-SiO_2_, suggesting higher crystallinity of this composite, which is consistent with DSC results (Table 5). Furthermore, T_β_ and Tγ were unaffected by silica. However, adding 5 wt.% of A-SiO_2_ to the PPO/PA11 blend increased β and γ relaxation temperatures. Moreover, the effect of A-SiO_2_ on the intensity of β and γ relaxation peaks varied to a similar extent as the tensile modulus, as can be seen in Table 4.

### 3.4. Mechanical Properties

Summarized mechanical properties of PPO, PA11, and PPO/PA11/A-SiO_2_ composites differing in silica type and content, including tensile and flexural properties, as well as notched Charpy impact strength, are shown in Table 4. PPO is a brittle polymer with high strength, whereas PA11 is a relatively tough material with ductility behavior [14,28,29]. It is clear from Table 4 that the mechanical behavior of the PPO/PA11 blend and its composites with silica was considerably different from that of blend components. The addition of 20 wt.% of PA11 to PPO significantly reduced the elongation at break. Moreover, impact strength decreased from 6 to 5 kJ/m^2^, which is three times lower, compared with PA11. This is because PPO is an amorphous polymer, whereas PA11 is a crystalline polymer, which is thermodynamically immiscible. As expected, tensile strength and moduli of the PPO/PA11 80/20 blend were lower than those of PPO and higher than those of PA11. However, great improvement in flexural strength, as well as in tensile and flexural modulus, was observed, suggesting significantly higher stiffness of the blend compared with PA11. It should be emphasized that the flexural strength of the blend was higher than that of PPO.

The dispersion state of silica particles, as well as their interaction with polymer matrix, can influence the properties of composites. The PPO/PA11/A-SiO_2_ composites had significantly higher tensile modulus than that of the PPO/PA11 blend and neat polymers, as shown in Table 4. This proves the strengthening effect of both polymer matrices. Tensile and flexural modulus, as well as tensile and flexural strengths, increased as a function of silica, and this fact, denoting a good dispersion of A-SiO_2_ particles, was also confirmed by SEM images, shown in Figure 4a and Figure 5. However, the effect of silica loading was moderate, suggesting that all composites exhibited a similar state of dispersion of A-SiO_2_. It is well known that finely dispersed silica interacts strongly with the polymer matrix, which results in a strengthening effect [41,43].

**Table 4:** Mechanical properties of PPO, PA11, and PPO/PA11/silica composites.

Sample	Tensile Modulus (MPa)	Tensile Strength (MPa)	Elongation at Break (MPa)	Flexural Modulus (MPa)	Flexural Strength (MPa)	Impact Strength (kJ/m^2^)
PPO	2515 ± 49	70 ± 1	10 ± 1	2250 ± 30	58 ± 1	6 ± 0.6
PA11	1240 ± 55	42 ± 1	299 ± 9	1180 ± 35	45 ± 1	15 ± 0.6
PPO/PA11	2192 ± 33	54 ± 1	7 ± 0.3	2080 ± 24	75 ± 1	5 ± 0.5
1 wt.% A-SiO_2_	4843 ± 53	60 ± 1	7 ± 0.2	2165 ± 28	86 ± 1	7 ± 0.3
5 wt.% A-SiO_2_	4998 ± 48	64 ± 1	7 ± 0.3	2322 ± 20	90 ± 1	6 ± 0.4
5 wt.% SiO_2_	2408 ± 48	56 ± 1	5 ± 0.2	2239 ± 55	77 ± 3	3 ± 0.3

Usually, a small amount of nanofillers increase impacts the strength of filled polymers. Improvement may be stronger when functionalized nanofillers are used between nanofiller and polymer functional groups, due to in situ chemical reactions [11,16,27,30]. However, at higher loading, nanofillers tend to agglomerate reducing impact strength. The addition of A-SiO_2_ increased impact strength as a result of compatibilization caused by a reaction between the amine group of silica and the terminal carboxyl group of PA11. Although impact strength decreased as silica content increased, it was still 20% higher than that of the blend without silica, as shown in Table 4. This phenomenon is most likely caused by the aggregation of silica and changes in the morphology depending on silica content (Figure 4a and Figure 5). The aggregates formed at higher A-SiO_2_ content behave like defects in the composites, inducing stress concentration and thus reducing the impact strength of the composites [7]. These results confirm the behavior observed in SEM. Moreover, no changes in elongation were observed for A-SiO_2_, regardless of its content.

The influence of neat silica on the strength and stiffness of PPO/PA11 was very small, compared with A-SiO_2_. This is probably due to the plasticization of the PPO phase, which was confirmed by the rheological behavior (Figure 7). As expected, the addition of 5 wt.% of neat silica decreased the impact strength and elongation at the breaking point of the blend, as often reported when nanoparticles were used [18,35,36]. A slight decrease in toughness can be caused by both the effect of viscosity reduction by plasticizing the viscous PPO phase and the agglomeration of silica particles [22,40,41,44].

### 3.5. Thermal Properties

**Figure 9:**
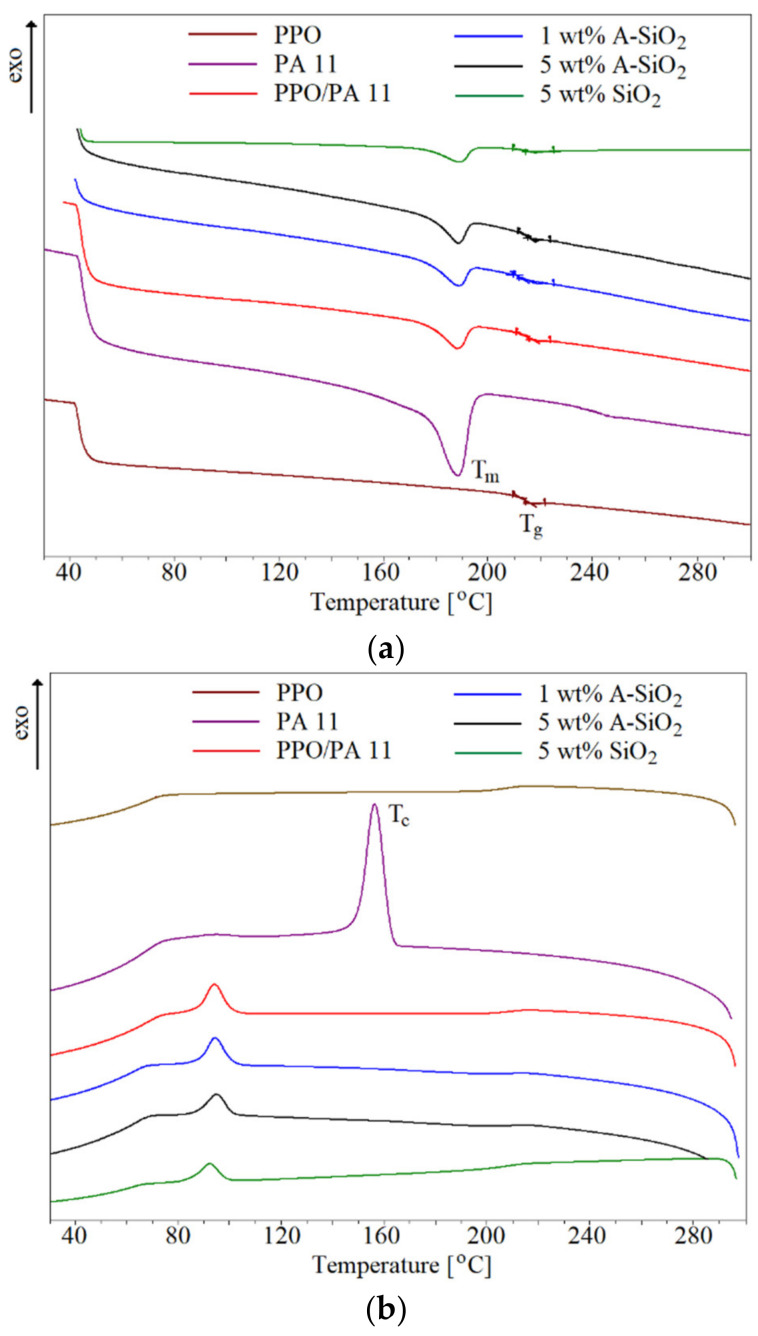
DSC curves of PPO, PA11, and PPO/PA11/silica composites: (**a**) second heating and (**b**) cooling.

It is well known that silica acts as a nucleating agent [10,30,37,39]. Therefore, the selective localization of silica can be proved by the crystallization behavior of composites. The thermal behaviors of PPO, PA11, and PPO/PA11/silica composites were investigated with DSC. The glass transition temperature (T_g_), melting temperature (T_m_), crystallization temperature, and the degree of crystallinity (T_c_ and X_c_, respectively) from the cooling of the PPO/PA11 blend and the composites were obtained from DSC analysis and are presented in Table 5 and Figure 9. In PPO/PA11 blend, T_g_ of the PPO phase, as well as T_m_ of the PA11 phase, were not affected by adding 20 wt.% of PA11 and 80 wt.% of PPO, respectively. However, a remarkable decrease in the crystallization temperature and crystallinity corresponding to the PA11 phase were observed. Indeed, T_c_ in the blend was 63 °C lower, compared with PA11. This indicates that these two polymers are highly immiscible, and the blend is incompatible [20,21,22]. The addition of A-SiO_2_ slightly increased the melting temperature and crystallization temperature, due to the nucleation effect of silica. These results are in good agreement with our previous study [11,30]. However, when the content of A-SiO_2_ increased to 5 wt.%, the melting and crystallization of the PPO/PA11/A-SiO_2_ composites shifted toward the lower values, suggesting the interaction between amine-functionalized silica particles and PA11. In addition, the higher value of the degree of super-cooling (T_m_–T_c_*)* indicates the induction times of polymer crystallization in the PPO/PA11 blend and in PPO/PA11/silica composites were higher than those in PA11 [11,36]. These results are in agreement with the T_c_ discussed above. The slight reduction in the crystallinity of the composites can be explained by an increase in viscosity [34]. Moreover, A-SiO_2_ can also act as a compatibilizer, improving the PPO and PA11 miscibility and reducing the crystallinity of PA11 [11]. Conversely, neat silica slightly increased the crystallinity of PA11, on the one hand, and decreased the crystallization temperature of PA11 and the glass transition temperature of PPO, on the other. This is probably due to the plasticization of the PPO phase, which was confirmed by the rheological behavior. These findings are consistent with SEM and DMTA results. It was reported that nanofillers can increase the crystallization rate and may cause a reduction in the degree of crystallinity [14,40,43,44]. 

## 4. Conclusions

The morphology, mechanical, and thermal properties of PPO/PA11/silica composites were investigated. With increasing A-SiO_2_ content, the morphology of PPO/PA11/A-SiO_2_ composites changed from droplet matrix to co-continuous with phase inversion. This phenomenon can be explained by the influence of A-SiO_2_ on the melt viscosity of the blend component and selective localization of A-SiO_2_ in the PA11 phase and consequent viscosity mismatch, as well as retarded mobility of the interface. Indeed, A-SiO_2_ was preferentially located in the PA11. By contrast, neat silica was mainly located in the PPO phase. This decreased the viscosity mismatch between the two phases, decreasing PPO viscosity; thus, the SiO_2_-rich PPO phase was completely elongated, forming a continuous phase. As a result, phase inversion was observed. The addition of modified silica significantly increased tensile and flexural moduli, as well as tensile and flexural strengths of the blend, due to the reinforcing effect. By contrast, at the same loading, significantly lower tensile and flexural properties were observed in the presence of SiO_2_ than A-SiO_2_ in the terms of differences in morphology. However, neat silica decreased impact strength and elongation at break, while A-SiO_2_ improved these properties. This improvement can be attributed to the strong link between amine-functionalized silica and the PA11 phase that induced stress transfer between the PA11 matrix and A-SiO_2_. Consequently, the storage modulus showed a positive correlation with the silica content. The addition of silica resulted in a higher glass transition temperature of the PA11 matrix and PPO dispersed phase in the composites, determined by DMTA. This improvement depends on the silica type and concentration. Moreover, A-SiO_2_ acted as a compatibilizer, improving the PPO and PA11 miscibility and reducing the crystallinity of PA11, without affecting the crystallization temperature, measured with DSC, while neat silica slightly increased the crystallinity of PA11 and decreased the crystallization temperature of PA11 and glass transition temperature of PPO. This is probably due to the plasticization of the PPO phase, which was confirmed by the rheological behavior. 

The applications of the obtained composites are similar to those of commercial grades of modified PPO, especially those working at high temperatures and in contact with superheated steam.

## 5. Patents

The results described in this manuscript are protected by Polish patent no 224 607.

## Figures and Tables

**Table 1:** Rheological data of PPO, PA11, and PPO/PA11/silica composites.

Sample	Gel Content (%)
PPO/PA11	0
1 wt.% A-SiO_2_	12
5 wt.% A-SiO_2_	18

**Table 2:** Rheological data of PPO, PA11, and PPO/PA11/silica composites.

Sample	η* (100 rad s^−1^) (MPa s)
PPO	21.3
PA11	3.3
PPO/PA11	6.3
1 wt.% A-SiO_2_	9.4
5 wt.% A-SiO_2_	11.0
5 wt.% SiO_2_	2.3

**Table 3:** DMTA data of PPO, PA11, and PPO/PA11/silica composites.

Sample	Temperature (°C)				Peak Intensity (MPa)			
	α_PPO_	α_PA 11_	β	γ	α_PPO_	α_PA 11_	β	γ
PPO	179	-	−18	−118	117	-	13.4	11.8
PA11	-	52	−69	−140	-	39.7	59.4	139
PPO/PA11	192	47	−77	−140	26.9	13.4	19.3	21.8
1 wt.% A-SiO_2_	194	55	−74	−139	29.7	18.7	22.9	27.5
5 wt.% A-SiO_2_	198	56	−72	−138	47.4	29.6	33.4	40.6
5 wt.% SiO_2_	195	58	−72	−138	29.6	18.6	22.5	27.4

**Table 5:** Thermal properties of PPO, PA11, and PPO/PA11/silica composites, measured using DSC.

Sample	T_g_ (C)	T_m_ (°C)	T_c_ (°C)	T_m_–T_c_ (°C)	X_c_ (%)
PPO	215	-	-	-	-
PA11	-	188	157	31	16.6
PPO/PA11	216	188	94	94	0.72
1 wt.% A-SiO_2_	216	190	96	94	0.68
5 wt.% A-SiO_2_	215	189	95	94	0.56
5 wt.% SiO_2_	212	189	92	97	0.59

## Data Availability

The data used to support the findings of this study are available from the corresponding author upon request.
